# Supplementary material for: Integrating an Extended-Gate Field-Effect Transistor in Microfluidic Chips for Potentiometric Detection of Creatinine in Urine
Source: Sensors (Basel). 2025 Jan 28;25(3):779. doi: 10.3390/s25030779 (PMC11820648; doi:10.3390/s25030779)
Supplement: Supplementary file 1 [file sensors-25-00779-s001.zip › sensors-3396919-supplementary.pdf]

Supplementary Materials

Supplementary Material S1: Microfluidic chip design and characterization.

Supplementary Material S2: Static and flow measurement setups.

Supplementary Material S3: Creatinine detection research results.

Supplementary Material S4: Capillary Electrophoresis test using the C^4D detector.

Supplementary S1: Microfluidic chip design and characterization.

We manufactured microfluidic devices using a Phrozen Sonic Mini 8 K 3D printer (China). The software used to create the 3D object was Fusion 360 Autodesk (San Rafael, EUA). The software used to control the 3D printing and slicing of the object was Chitubox version 1.8 and the resin used for printing was Anycubic Clear (405 nm). The device model manufactured featured a single-channel structure with sample inlet and outlet, as shown in Figure S1 below, which allowed for the efficient delivery of liquid to the electrodes that formed the side walls of the channel.

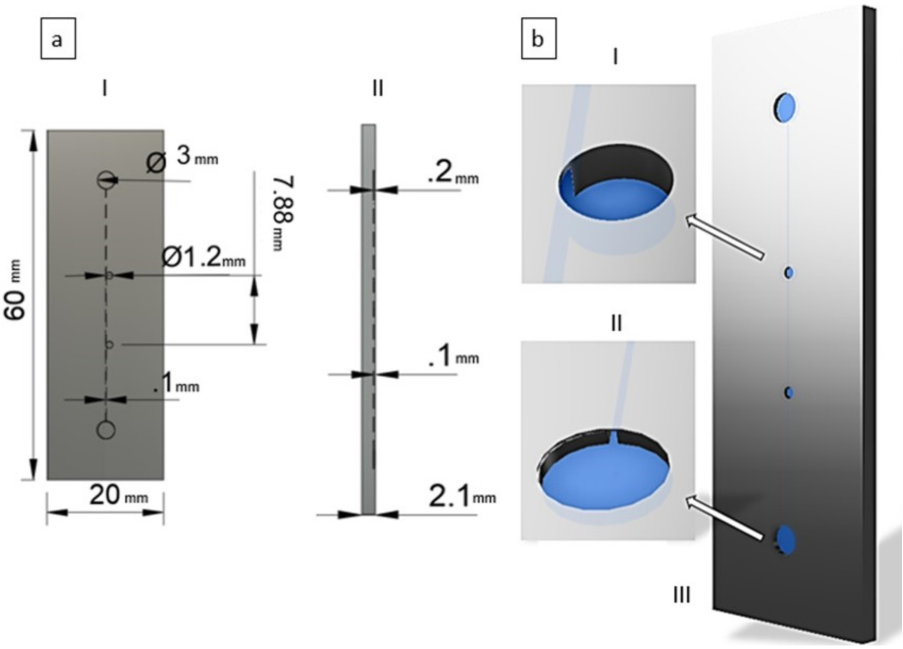

Figure S1: Rendering of the computer drawing in Fusion 360 (version 2.02.0.17954, Educational License).

The flow measurement device in Figures S1a and S1b was the first to be developed. It was 60 mm long, 20 mm wide, and 2.1 mm high. There was only one flow channel projected onto the chip, which had a computerized projection 50 mm long, 0.200 mm deep, and 0.100 mm wide. There were circular reservoirs at each end of the channel with a diameter of 3 mm and a depth of 0.200 mm. Along the fluid channel, there were two circles 1.20 mm in diameter and 0.200 mm deep. They were used as an inlet for the working and reference electrode, 7.88 mm apart. A thin film of 0.1 mm sealed the channel. The process of printing the device on the 3D machine took 24 minutes.

An Axio Observer A1 inverted microscope (Zeiss, Germany) equipped with a HAL 100 light source for optical analysis was used to check the dimensions of the microfluidic chip channels after they had been printed on the 3D machine.

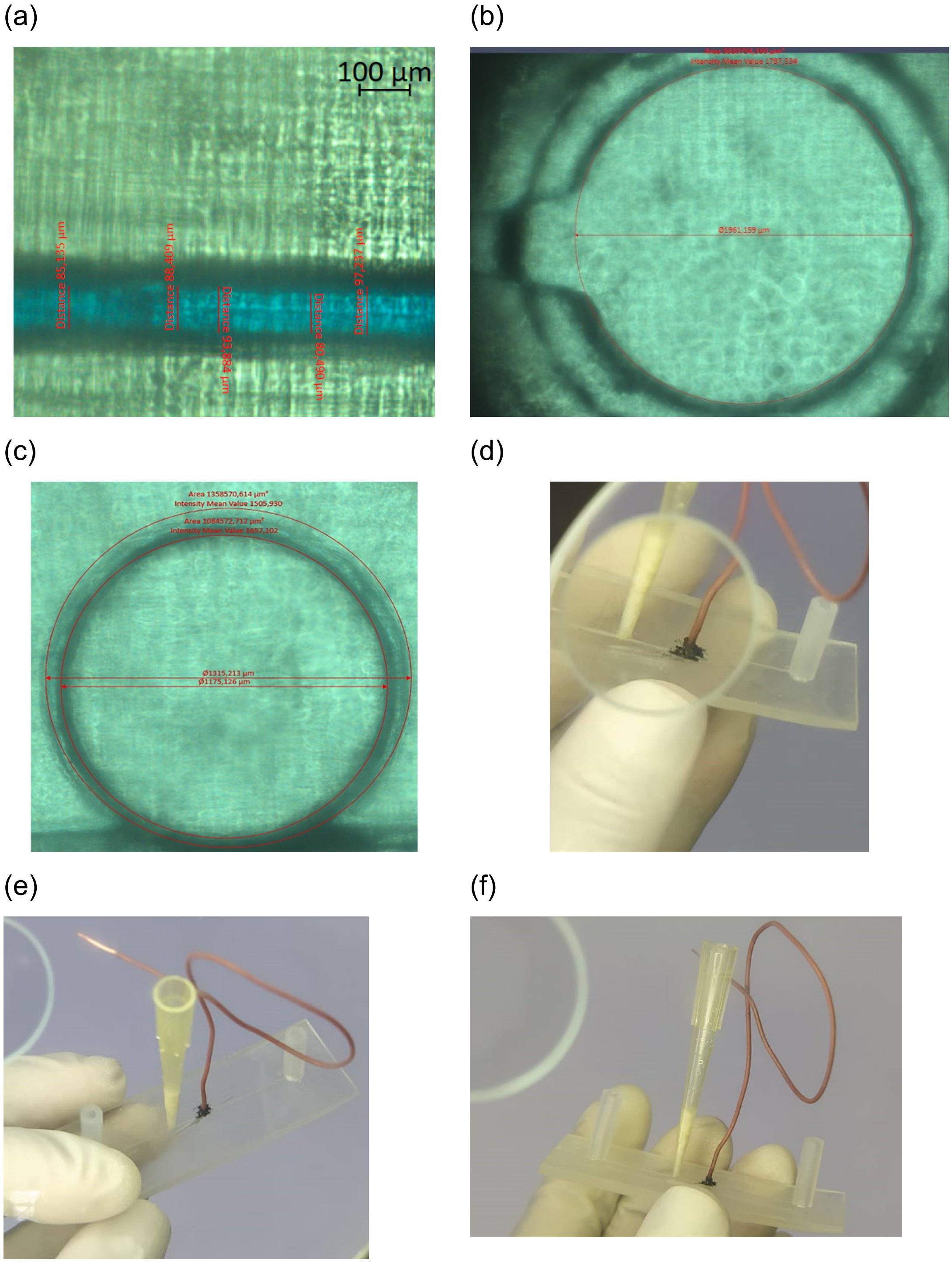

Figure S2: Microfluidic chip. (a) channel dimensions. (b) reservoirs dimensions. (c) electrode hole. (d) zoom in reference electrode and indicator electrode on the channel. (e) Image above of microfluidic device. (f) lateral image of microfluidic chip.

When characterized, the chip had channel dimensions of 99.66 x 176.34 μm; with 131.52 mm for the electrode inlet and 196.11mm inside the reservoirs.

As we were interested in ensuring that the interaction with the electrode could be carried out without other contributions, such as turbulence in the flow, the single, straight channel chip allowed for the adequate flow of the particles that made up the samples.

After characterizing the print, the reference and working electrodes were carefully attached to the chip channel to form a lateral detection wall. Finally, the chip was finished by connecting Tygon® tubes to the inlet and outlet reservoirs. Tygon® tubes had an outer diameter of 4 mm and a length of 170 mm. A 60 x 25 mm injection needle was connected to the tubes for attaching the syringe pump.

Supplementary Material S2: Static and flow measurement setups.

The static measurement system, shown in Figure S3 below, consisted of a reference electrode and an indicator electrode positioned inside a 10 mL beaker

containing thaw analysis solution. For the flow measurements, the reference and indicator electrodes were positioned in pre-designed holes in the microfluidic chip. A syringe pump was used to introduce the samples into the chip's channel which, after traveling along the flow path to the other end of the channel, were collected in a 5 mL beaker. This is shown in Figure S3 below.

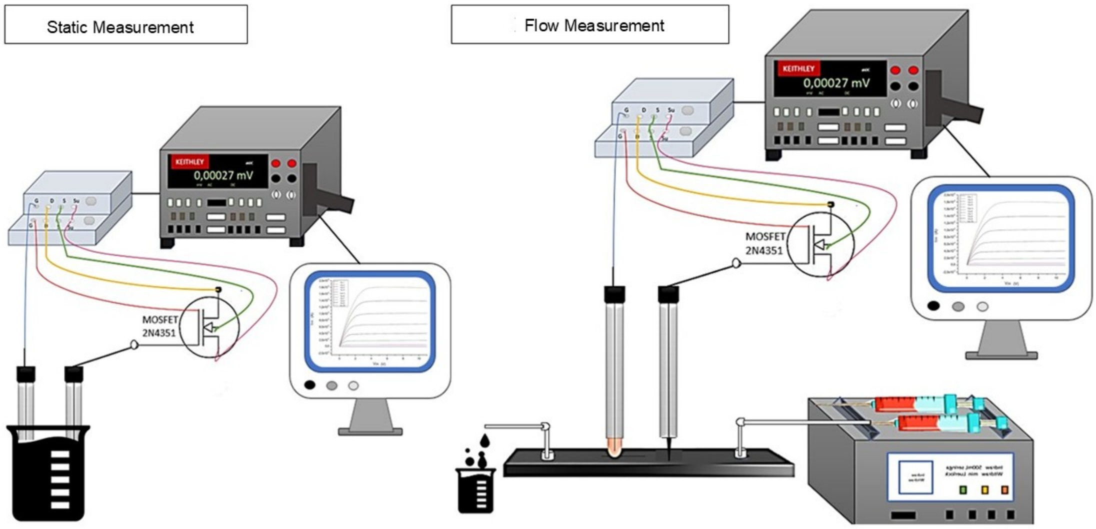

Figure S3: Static and dynamic measurement systems.

In both the static and flow systems, the reference electrode was connected directly to an electrical unit (SMU) to receive the signals from the Semiconductor Parameter Analyzer (KI 4200 MPSMU, Keithley USA). The indicator electrode was connected to the port terminal of the Motorola commercial MOSFET model 2N4351 and also connected to the electrical unit for contact with the analyzer. A computer monitor integrated into the analyzer projected and allowed access to the data generated during the measurements.

The probe stations and semiconductor parameter analyzers were calibrated regularly to ensure the accuracy of data recording and analysis. The extended gate comprising the EGFET was connected externally to the commercial MOSFETs via the SMU. The flow detection platform, as shown in Figure 1 above, continuously delivered the sample via syringe pump to the channel of the chip where the gate extension of the FETs was located.

Supplementary Material S3: Published data regarding creatinine detection.

Method/Material	LOD (μmol L^{-1})	Sample	Real range (μmol L^{-1})	Ref.

<table>
<tr><td>Potentiometry / PVC-o-NPOE - OMCP</td><td colspan="2">0.95</td><td>Human urine</td><td>1.0 – 10000</td><td>49</td></tr>
<tr><td>Colorimetric/ PDMS-PMMA-Urease</td><td colspan="2">(Flow test) 0.8842</td><td>Human blood</td><td>3.5368 – 707.36</td><td>51</td></tr>
<tr><td>SSNE1 / PET-PEI-CD-PEI</td><td colspan="2">(Flow test) 0.005</td><td>Human urine</td><td>0.05-100</td><td>52</td></tr>
<tr><td>Colorimetric / AuNPs+APT</td><td colspan="2">870</td><td>Synthetic urine</td><td>2000-20000</td><td>53</td></tr>
<tr><td>Voltammetry / CuNWs-SPE</td><td colspan="2">10</td><td>Buffer</td><td>50 – 500</td><td>54</td></tr>
<tr><td>SERS / Ag NCs-C-CNF</td><td colspan="2">0.5-5</td><td>Human urine</td><td>0.5-5</td><td>55</td></tr>
<tr><td>Colorimetric / EDTA-AuNPs</td><td colspan="2">125</td><td>Synthetic urine</td><td>2500 - 10000</td><td>56</td></tr>
<tr><td>Voltammetry / ePADs</td><td colspan="2">5</td><td>Blood and urine</td><td>30 – 45000</td><td>57</td></tr>
<tr><td>Colorimetric / Ag NPs</td><td colspan="2">0.00690</td><td>Saliva artificial</td><td>0.01–0.06</td><td>58</td></tr>
<tr><td>Fluorescence / PSS-CuNCs</td><td colspan="2">0.00248</td><td>Water</td><td>2.0-24.0</td><td>59</td></tr>
<tr><td>Colorimetric / AuNPs</td><td colspan="2">0.084</td><td>Synthetic urine</td><td>1–50</td><td>60</td></tr>
<tr><td>Voltammetry / GCE- SPCE</td><td colspan="2">6.6</td><td>Synthetic urine</td><td>50 – 650</td><td>61</td></tr>
<tr><td>Colorimetric / C-AuNPs</td><td colspan="2">26.5</td><td>Synthetic urine</td><td>26.5–70.7</td><td>62</td></tr>
<tr><td>Colorimetric / GNPs@N-CDs</td><td colspan="2">0.1768</td><td>Human urine</td><td>0.0009 – 0.09</td><td>63</td></tr>
<tr><td>Colorimetric / UiO-66 metal–organic framework</td><td colspan="2">17.684</td><td>Water</td><td>----</td><td>64</td></tr>
<tr><td>Fluorescence / UE^{3+}:PQDs SrV</td><td colspan="2">0.016</td><td>Human urine</td><td>0.5–100</td><td>65</td></tr>
<tr><td>Electrochemiluminescence / N-CQDs</td><td colspan="2">0.0087</td><td>Human urine</td><td>0.01 - 10</td><td>66</td></tr>
<tr><td>Colorimetric / μPADs</td><td colspan="2">(Flow test) 5428.988</td><td>Synthetic urine</td><td>442.1 – 44210</td><td>67</td></tr>
<tr><td>Voltammetry / ITO</td><td colspan="2">28</td><td>Human blood</td><td>50 – 2000</td><td>68</td></tr>
<tr><td rowspan="4">Potentiometry / Res-OMCP-KTPB-Graphite</td><td rowspan="2">4910</td><td>Flow</td><td rowspan="2">Water</td><td rowspan="4">3000 - 24000</td><td rowspan="4">This work</td></tr>
<tr><td>6430</td></tr>
<tr><td>5520</td><td>5250</td><td>Synthetic urine</td></tr>
<tr><td>6320</td><td>1310</td><td>Buffer</td></tr>
</table>

Supplementary S4: Capillary Electrophoresis test using the C^4D detector.

The test was an adaptation of a previous study [69]. A fused silica capillary of 50 µm i.d. and 375 µm o.d. obtained from Agilent (Brazil) with a total length of 53 cm and an effective length from the inlet to the detector of 45 cm was initially conditioned: 15 minutes with 1 mol L^{-1} NaOH solution; 15 minutes with deionized water; 6 minutes with concentrated HCl, and 30 minutes with electrolyte. The electrolyte consisted of 40 mmol L^{-1} of MES and 20 mmol L^{-1} of L-His. The temperature of the capillary was controlled at 25 °C. Data acquisition was carried out using LabView software version 20.0.1. The separation voltage was +20 kV and the hydrodynamic injection of the sample was programmed for six seconds (at 11 kPa).

The sample concentrations ranged from 3 mmol L^{-1} to 9 mmol L^{-1}, 15 mmol L^{-1}, 21 mmol L^{-1}, 24 mmol L^{-1}, and 27 mmol L^{-1} of creatinine. The matrices were deionized water and commercially purchased synthetic urine. To use the synthetic urine, we first ran tests with 5-, 10-, 20- and 100-fold dilutions and then prepared the samples in the 10- and 100-fold diluted urines.

The results from the Capillary Electrophoresis test using the C^4D detector for creatinine samples prepared in water, urine, and buffer can be seen in Figure S4 below.

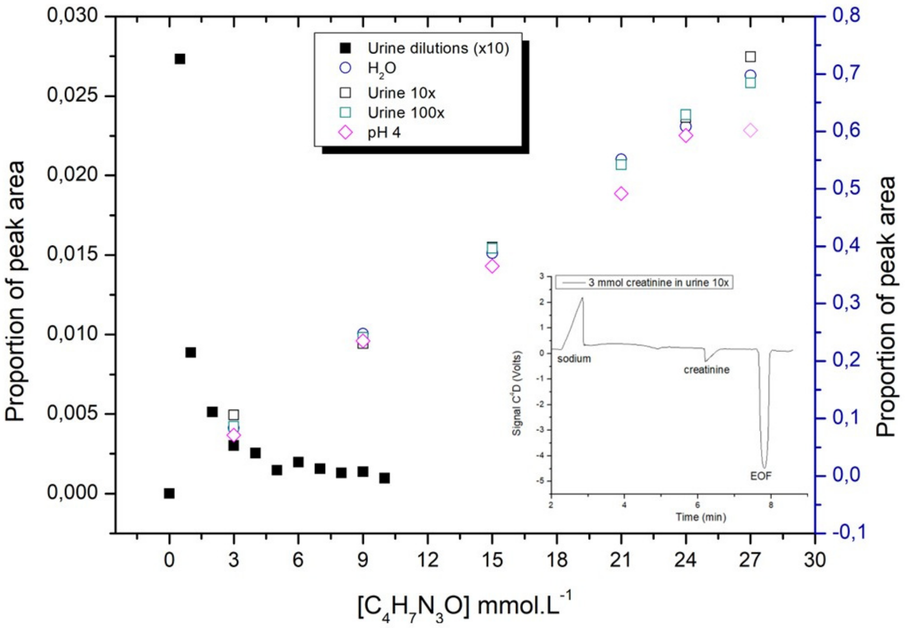

Figure S4: Graph of the integration of creatinine peak areas for synthetic urine diluted 5 to 100 times and concentrations of 3, 9, 15, 21, 24, and 27 mmol L^{-1} creatinine in water, pH 4 buffer, 10x, and 100x diluted synthetic urine used as matrices. The electropherogram of the serum creatinine sample prepared in 10x diluted urine with a concentration of 3 mmol L^{-1} is shown at the bottom right, with the first peak being sodium, the second creatinine, and the largest the electrometric flow.

As can be seen in Figure S4, synthetic urine could be used without the need for dilution since; at the fifth dilution it showed a peak area three times smaller than the lowest concentration (3 mmol L^{-1}) tested for all the matrices used in this research.

This can be reaffirmed by the behavior of the concentration lines of the solutions in the matrices. These only show the behavior of the creatinine concentrations added to the water, urine, and buffer matrices. Mathematically, by comparing the slopes of the measurements, we realized that these values were equivalent. Even so, in order to reduce the influence of the concentration of other species present in the urine, such as the presence of sodium, we decided to use ten times diluted urine for the sensor tests.

For the tests using capillary electrophoresis, the LOD for the water measurement was 1.1 mmol L^{-1}, and the LOD for the urine measurement was 0.9 mmol L^{-1}, while the LOD for the buffer measurement was 1.8 mmol L^{-1} and the LOQs were 3.7 mmol L^{-1}, 2.9 mmol L^{-1}, and 6.0 mmol L^{-1}, respectively.

The LODs and LOQs obtained with the capillary electrophoresis test were lower than those obtained with the sensor developed. However, considering the portability of the system, the sensor is more suitable for portable point-of-care systems.
